# Insights into organizational health literacy in Italian hospitals: findings from the M-POHL network project

**DOI:** 10.1093/heapro/daaf137

**Published:** 2025-08-25

**Authors:** Chiara Lorini, Luigi Palmieri, Brigid Unim, Salvatore Zimmitti, Carla Lunetta, Claudia Biagi, Francesco Toccafondi, Patrizio Zanobini, Simone Iadevaia, Maria Gabriella Cacciuttolo, Camilla Lombardo, Benedetta Marcozzi, Angela Ancona, Andrea Paladini, Daniela Galeone, Maria Lucia Specchia, Guglielmo Bonaccorsi

**Affiliations:** Department of Health Sciences, Health Literacy Laboratory, University of Florence, viale GB Morgagni 48, Florence 50134, Italy; Department of Cardiovascular, Endocrine-Metabolic Diseases and Aging, Istituto Superiore Di Sanità, Viale Regina Elena, 299 - 00161 Roma, Italy; Department of Cardiovascular, Endocrine-Metabolic Diseases and Aging, Istituto Superiore Di Sanità, Viale Regina Elena, 299 - 00161 Roma, Italy; Medical School of Specialization in Hygiene and Preventive Medicine, University of Florence, viale GB Morgagni 48, Florence 50134, Italy; Medical School of Specialization in Hygiene and Preventive Medicine, University of Florence, viale GB Morgagni 48, Florence 50134, Italy; Department of Health Sciences, Health Literacy Laboratory, University of Florence, viale GB Morgagni 48, Florence 50134, Italy; Medical School of Specialization in Hygiene and Preventive Medicine, University of Florence, viale GB Morgagni 48, Florence 50134, Italy; Department of Health Sciences, Health Literacy Laboratory, University of Florence, viale GB Morgagni 48, Florence 50134, Italy; Medical School of Specialization in Hygiene and Preventive Medicine, University of Florence, viale GB Morgagni 48, Florence 50134, Italy; Department of Life Sciences and Public Health, Università Cattolica del Sacro Cuore, Largo Francesco Vito, 1, 00168 Roma, Italy; Scientific Communication Unit, Istituto Superiore di Sanità, Viale Regina Elena, 299 - 00161 Roma, Italy; Department of Cardiovascular, Endocrine-Metabolic Diseases and Aging, Istituto Superiore Di Sanità, Viale Regina Elena, 299 - 00161 Roma, Italy; Scientific Communication Unit, Istituto Superiore di Sanità, Viale Regina Elena, 299 - 00161 Roma, Italy; Department of Life Sciences and Public Health, Università Cattolica del Sacro Cuore, Largo Francesco Vito, 1, 00168 Roma, Italy; General Directorate for Health Prevention, Ministry of Health, Viale Giorgio Ribotta, 5, 00144 Rome, Italy; Department of Life Sciences and Public Health, Università Cattolica del Sacro Cuore, Largo Francesco Vito, 1, 00168 Roma, Italy; Department of Health Sciences, Health Literacy Laboratory, University of Florence, viale GB Morgagni 48, Florence 50134, Italy

**Keywords:** hospital, settings approach, interdisciplinary evaluation, self-assessment, healthcare equity, health information access, organizational health literacy

## Abstract

Hospitals are recognized as settings for health promotion, serving as a process that empowers individuals to gain greater control over and improve their health. Health-literate organizations play a crucial role in health promotion by creating supportive environments that ensure equitable access to health information and services, empowering individuals to engage with, understand, evaluate, and apply health information through diverse communication channels and social resources. The International Self-Assessment Tool for Organizational Health Literacy of Hospitals (OHL-Hos) was used for the first time in Italy to evaluate the implementation of OHL principles in two local hospitals and one academic hospital. The OHL-Hos is organized into 8 standards, 21 substandards, and 155 items. The degree of fulfillment with the OHL principles was calculated for substandards and standards to assess their accomplishment within the organization, along with an overall score. In each hospital, the self-assessment was carried out by an interdisciplinary team; the team members initially conducted the assessment individually, and then a joint assessment was performed to reach a consensus. The two local hospitals presented similar results and an overall level of OHL responsiveness (41.62% and 39.15%, respectively) lower than the academic hospital (63.22%). The OHL principles were found to be only partially addressed and fulfilled. The OHL-Hos tool proved valuable in identifying the most critical areas that require targeted interventions, aimed at enhancing both the OHL of the organizations and the health of individuals as a result.

Contribution to Health Promotion

- Redefining determinants of health through innovative perspectives, as advocated by the Ottawa Charter, shifts the focus from disease treatment to prevention and health promotion.
- Upholding relevance through health advocacy, enabling, and mediation ensures that health promotion strategies remain effective and adaptable to evolving public health challenges.
- Hospitals serve as key settings for health promotion by integrating prevention and wellness initiatives into patient care and hospital policies.
- Creating health-promoting environments in hospitals enhances the well-being of patients, staff, and the broader community.
- The OHL-Hos tool proved valuable in identifying the most critical areas that require targeted interventions.

## INTRODUCTION

### Hospitals as settings for health promotion

The Ottawa Charter introduced a new perspective on public health by emphasizing rethinking how determinants of health are conceptualized, described, and analyzed, as well as by proposing innovative methods for solving public health challenges (WHO 1986, 2021). The five strategies described in the Charter—building healthy public policy, creating supportive environments for health, strengthening community action, developing personal skills, and reorienting health services—have shaped the framework for addressing public health issues for decades (WHO 2021).

Following the Ottawa Charter principles, hospitals are “settings for health promotion,” such as emphasizing the importance of reorienting health services not just toward treatment but also toward actively promoting health and equity. This requires creating supportive environments by improving organizational structures, policies, and communication systems to facilitate access to healthcare and promote health. In this context, health promotion in hospitals is seen as a process that empowers people to increase control over and to improve their health. In fact, health-promoting hospitals aim to go beyond curing illnesses, also focusing on fostering the health and well-being of patients, staff, and the broader community (Pelikan *et al*. 2001). In doing so, health-promoting hospitals have an implicit salutogenic orientation, focusing on a comprehensive concept of health and on the mobilization of resources for health, as well as on reducing risk factors associated with diseases for a wide set of target groups, irrespective of their positioning along the health-disease continuum (Dietscher *et al*. 2016).

### Organizational health literacy

The 2021 World Health Organization (WHO) Health Promotion Glossary of Terms defined health literacy (HL) as “the personal knowledge and competencies that accumulate through daily activities, social interactions and across generations.” In the glossary, it is stressed that “personal knowledge and competencies are mediated by the organizational structures and availability of resources that enable people to access, understand, appraise and use information and services in ways that promote and maintain good health and well-being for themselves and those around them” (WHO 2021). This is the result of the most recent evolution of the concept of HL toward a relational model, a balance between individual and contextual skills or demands (Sørensen *et al*. 2019). In fact, HL is now considered as a social practice that involves individuals, communities, organizations, public policies, and societies (Samerski 2019, WHO 2022). In such a perspective, HL is not the sole responsibility of the individuals, but also of all information providers. Consequently, to guarantee the right to health, the health information system needs to develop the capability to meet the complex demands of people (Bonaccorsi and Lorini 2024). In fact, HL responsiveness is a responsibility of different stakeholders, as it is defined as the ability of healthcare professionals, services, systems, organizations, and policymakers—both within and across government sectors—to acknowledge and address the diverse traditions, strengths, needs, and preferences related to HL. HL responsiveness consists in the provision of services, programs, and information in ways that promote equitable access to health information and services, foster meaningful engagement, help people access, comprehend, evaluate, recall, and apply health-related information through verbal, written, digital, and other communication channels, as well as social resources, and promote the health and well-being of individuals, families, groups, and communities (Osborne *et al*. 2022).

The concept of organizational health literacy (OHL) describes an organization-wide effort to transform organization and delivery of care and services to make it easier for people to navigate, understand, and use information and services to take care of their health (Farmanova *et al*. 2018). The founding principles of OHL—making health services more navigable and patient-friendly, as well as developing personal skills and creating supportive environments—are perfectly in line with the Ottawa Charter principles, and with its extension to the Health Promoting Hospitals. In particular, OHL supports health-promoting hospitals, as it simplifies communication, reducing barriers to care and creating environments where people of all literacy levels can navigate the healthcare system. This perfectly aligns with the health-promoting hospital goal of improving health outcomes through education, empowerment, and supportive environments.

In a discussion paper published by the Institute of Medicine of the National Academies in 2012, Brach defined a Healthcare Organization as Health-Literate when it programs and implements strategies to facilitate easier navigation for people, irrespective of their level of HL. This is achieved by providing clear information for all individuals, facilitating their navigation of health-related information and services and empowering them to make informed decisions. The elimination of existing barriers in these areas is also crucial (Brach *et al*. 2012). The author provided a comprehensive list of 10 attributes that define a healthcare organization with HL capabilities. These attributes have been shown to facilitate the establishment of a healthcare system that is person-centered, evidence-based, and quality driven (Farmanova *et al*. 2018).

The Vienna WHO Collaborating Centre for Health Promotion in Hospitals and Healthcare, in collaboration with the Austrian Network of Health Promoting Hospitals and Healthcare Institutions, developed the “Vienna Concept of Health-Literate Hospitals and Healthcare Organizations” (V-HLO). In this model, OHL is more explicitly linked to health promotion than in the framework proposed by Brach *et al*. (2012), particularly in relation to the settings approach developed for health-promoting hospitals (Pelikan *et al*. 2005). Rather than focusing on a list of attributes, the V-HLO uses a matrix model that includes not only patients as stakeholders but also organizational staff and the regional population. Beyond the healthcare domain, in such a model, OHL is connected to aspects like accessing, living in, or working within the hospital, as well as disease prevention and health promotion. The scope of the model expands from merely addressing patients’ existing HL to enhancing the personal HL of all stakeholders, supporting disease management, prevention, and lifestyle improvements. The V-HLO adopts the comprehensive definition of HL from the HLS-EU Consortium (Sørensen *et al*. 2012), which defines HL as the ability to find, understand, evaluate, and apply health-related information for decision-making in daily life regarding healthcare, disease prevention, and health promotion. Within the V-HLO, HL is not only seen as a fundamental element of health promotion but, like health promotion itself, as a core component of healthcare quality (Pelikan 2019).

In 2018, M-POHL (Action Network on Measuring Population and Organizational Health Literacy) was established to strengthen HL in the WHO European Region by providing high-quality, internationally comparable data to inform evidence-based policies and targeted interventions. According to its Concept Note and the Vienna Statement, M-POHL aims to

- foster collaboration between research and policy,
- address HL among the general population and patients,
- institutionalize regular international surveys on population HL,
- promote HL-friendly systems and organizations,
- support the assessment of OHL, and
- advance evidence-based policy and practice.

To date, M-POHL implemented three projects with a timeline from 2023 to 2027:

- Project 1: Health Literacy Survey 2024–26.
- Project 2: Assessing Organizational Health Literacy.
- Project 3: Evidence-based Policy and Practice.

As a goal of the Project 2, and based on the V-HLO framework, M-POHL piloted the International Self-assessment Tool for Organizational Health Literacy (responsiveness) of Hospitals (OHL-Hos), through a participatory process (International Working Group Health Promoting Hospitals and Health Literate Healthcare Organizations 2019, Finbråten *et al*. 2024). The OHL-Hos tool can be used by a variety of professionals and teams within healthcare organizations: presidents and chief executive officers, program directors, quality management and human resources development staff, health promoters in hospitals. Moreover, it is suitable for organizations at any stage of engagement with HL (both beginners and those already implementing initiatives), looking to diagnose their current status, plan and implement improvements, and monitor progress in OHL.

To date, six countries (Austria, Czech Republic, Germany, Italy, Norway, and Serbia) have translated, culturally adapted tool, and piloted the tool in one or more hospitals. However, the results of these pilot studies have not yet been published.

### Aim

The aim of this study is to evaluate the extent to which OHL principles are implemented in a sample of Italian hospitals, using the OHL-Hos tool. As this research represents the first attempt to apply the OHL-Hos tool in Italy, this study does not aim to be representative of the overall Italian healthcare system but rather to provide initial insights.

## MATERIALS AND METHODS

### Study design

The study was proposed to a convenience sample of three hospitals located in central Italy, which accepted to participate. In each hospital, the OHL self-assessment process was conducted within an interdisciplinary and interhierarchical framework to ensure that diverse perspectives were adequately represented. The methodology included seven key steps, as defined by the M-POHL network and reported in the OHL-Hos:

1. obtain a self-assessment mandate from the responsible management of the unit or organization and clarify the scope of the assessment,
2. the management has to appoint a person to coordinate the self-assessment,
3. identification of the assessment team,
4. the members of the assessment team perform individual assessments,
5. collecting documents if possible,
6. the assessment team perform a joint assessment, and
7. selection and implementation of improvement measures.

With regard to Point 3, a team of 5–10 individuals had to be assembled, including representatives from management, quality management, health promotion, human resources, clinical and nonclinical professions (e.g. medicine, nursing, therapeutic roles), technical services, patient representatives, and communications. Members were chosen to ensure different perspectives in the organization: they worked in different areas of the hospitals, had different backgrounds, but had detailed knowledge of the hospital due to their roles and responsibilities (e.g. head of nurses, booking center representatives, quality managers, hospital management doctors). The evaluation therefore requires the involvement of expert personnel with specific responsibilities within the organization (e.g. management, quality management, health promotion, human resource development, nursing, other healthcare professionals, building services, communications).

As described previously, team members performed an individual evaluation using the OHL-Hos tool by reviewing each item based on their professional perspective and, subsequently, the team developed a joint assessment through a series of deliberations and reconciliations of the individual evaluations, which culminated in a consensus. The joint assessment leverages the strengths, perspectives, and expertise of multiple stakeholders, thereby producing a more comprehensive and nuanced evaluation. Collaborative efforts ensure that all relevant factors are systematically considered, resulting in an assessment that is accurate, balanced, and reflective of diverse viewpoints. Furthermore, joint assessments foster transparency and interprofessional cooperation, ultimately enhancing decision-making processes and facilitating more effective problem-solving.

The assessment was conducted from September 2023 to December 2024.

The study was conducted according to the Helsinki declaration.

### Characteristics of the hospitals included in the study

The three hospitals were chosen using convenience criteria. They were all public, non-for-profit, general, and acute facilities. Two were general, local hospitals situated in small cities, while one was an academic hospital placed in a large city. The two general hospitals were similar in terms of the number of healthcare workers employed, as well as the types and the activity volume, while the academic hospital presented a higher number of healthcare workers employed and of activity volume. Vocational training was offered by all three hospitals, both in the form of continuous training and specialist training, while the academic hospital also offered academic training (Table 1).

**Table 1.** General characteristics of the hospitals included in the study.

Characteristics	Local Hospital 1	Local Hospital 2	Academic hospital
Catchment area	City (≥15 000 and <100 000 inhabitants)	City (≥15 000 and <100 000 inhabitants)	Large city^a^ (≥100 000 and <1 000 000 inhabitants)
Number of physicians	307	292	1065
Number of hospital admissions in 2023	16 071	15 773	56 389
Number of day hospitals in 2023	2499	2337	14 491
Surgical DRG in 2023	6630	5946	33 686
Medical DRG in 2023	9441	9827	22 703
Main areas of expertise of the organization	General and acute care hospital	General and acute care hospital	General and acute care hospital
Type of organization (i.e. for profit or not for profit, public or private)	Public not for profit	Public not for profit	Public not for profit
Type of vocational training (i.e. continuous training, academic training, specialist training)	Continuous training, specialist training	Continuous training, specialist training	Continuous training, academic and specialist training

^a^This refers to the city where the hospital is located, instead of the catchment area, because in Italy, academic hospitals have a national-level perspective.

### Measurement tool and data analysis

The study was conducted using the Italian version of the international self-assessment tool OHL-Hos. The original OHL-Hos tool (developed in English) was translated in the Italian language by two independent research groups and then back-translated in English. Each group was composed by four researchers (including a mother-tongue and a professional translator). The two groups reached a consensus in the definition of the draft of the tool: the consensus process was performed during six periodic meetings, lasting at least 2 h. During the meetings, all discrepancies and comments rising from the two translations were discussed and solved. In case of disagreement, the principal investigator of the project and the professional translator took the final decision.

Then, the draft of the tool was subjected to a cultural adaptation process for the Italian context by the third research group. The cultural adaptation process group consisted of 11 experts from diverse backgrounds, including medicine, public health, nursing science, and community health. Their roles varied and included medical residents, professors, researchers, PhD students, and a hospital-based nurse. During the adaptation process, less familiar terms were replaced with more appropriate alternatives; examples were added to clarify the meaning of certain items; and different terms were selected to refer to structures or services not found in Italian hospitals, while preserving the original meaning or referring to similar services. In the glossary, where applicable, references were replaced with comparable Italian projects or tools. At the end of the cultural adaption process, a draft of the tool was proposed and discussed in a consensus meeting involving the three research groups. As a result of the consensus meeting, the final version of the Italian version of the OHL-Hos was defined. The number and content of the items remain unchanged between the original and the Italian versions of the tool.

The tool is structured into 8 standards, 21 substandards, and 155 items. The items operationalize elements that are concretely observable or measurable and classify the self-assessed level of compliance within the organization. For each item, four categories of fulfillment are defined (response options) as follows: completely fulfilled (76%–100%), fulfilled to a larger extent (51%–75%), fulfilled to a lesser extent (26%–50%), or not fulfilled (0%–25%). An additional response option is considered to indicate that a specific item is not applicable to the organization. For each response option, a score is associated, from 0 (not fulfilled) to 3 (completely fulfilled). To evaluate the extent to which each substandard was met, the number of applicable items for each hospital was considered, along with the maximum achievable points. The degree of fulfillment was then calculated by comparing the points achieved with the total possible points. The standardization process entailed the equal evaluation of each substandard for the calculation of the degree of fulfillment of the corresponding standard, irrespective of the number present of items. This principle was also applied to the overall score calculation, where each standard was assigned equivalent weight.

## RESULTS

Table 2 reports the main characteristics of the evaluation process conducted in the three hospitals. The number of people involved in the self-assessment ranged from 9 to 14; each participant was involved in both the individual and in the joint assessment evaluation. The teams were composed of different professionals with different responsibilities and expertise.

**Table 2.** Main characteristics of the evaluation process in the included hospitals.

Characteristics	Local Hospital 1	Local Hospital 2	Academic hospital
Part of the organization for which you conduct the self-assessment	Whole organization	Whole organization	Whole organization
Number of people involved in the individual self-assessment	9	10	14
Number of people involved in the joint assessment	9	10	14
Professional figures involved in the assessment	Physicians (*N* = 2), nursing staff (*N* = 2), other health professions (*N* = 2), management and administration (*N* = 2), other staff (Journalist Press Office, *N* = 1)	Physicians (*N* = 2), nursing staff (*N* = 2), other health professions (*N* = 2), management and administration (*N* = 2), other staff (patients’ guarantor for the right to health, *N* = 1; building maintenance, *N* = 1)	Physicians (*N* = 3), nursing staff (*N* = 3), other health professions (*N* = 2), management and administration (*N* = 3), other staff (public relations office and claims management committee, *N* = 2; building maintenance, *N* = 1)

**Figure 1.**
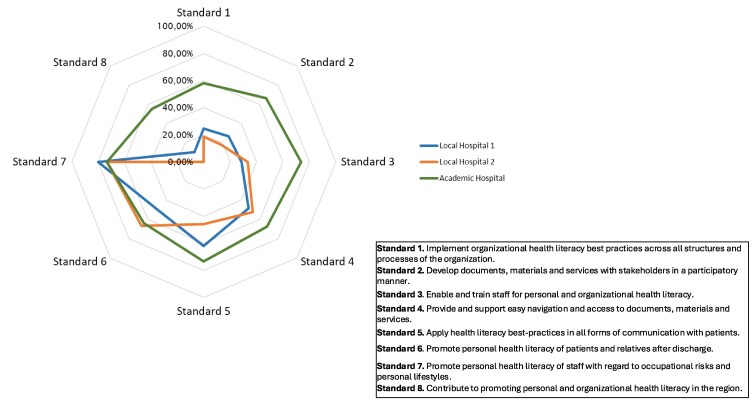
Overview of the fulfillment of the eight standards in the three hospitals included in the study.

The results by standards and substandards are reported in Fig. 1 and Table 3.

**Table 3.** Results of the OHL assessment: degree of fulfillment based on the joint assessment for each participating hospital.

Standards and sub-standards	Local Hospital 1 (degree of fulfillment %)	Local Hospital 2 (degree of fulfillment %)	Academic hospital (degree of fulfillment %)
Standard 1: Implement organizational health literacy best practices across all structures and processes of the organization	24.44	18.57	57.94
Sub-standard 1.1 (5 items): The management of the organization is committed to implementing, monitoring, and improving organizational health literacy	6.67	6.67	40.00
Sub-standard 1.2 (7 items): The organization makes organizational health literacy an organizational priority and secures adequate infrastructures and resources for implementing it	33.33	19.05	57.14
Sub-standard 1.3 (10 items): The organization ensures the quality of organizational health literacy interventions by quality management	33.33	30.00	76.67
Standard 2: Develop documents, materials, and services with stakeholders in a participatory manner	26.67	18.33	66.67
Sub-standard 2.1 (5 items): The organization involves patients in the development and evaluation of patient-oriented documents, materials, and services	20.00	20.00	66.67
Sub-standard 2.2 (2 items): The organization involves staff in the development and evaluation of staff-oriented documents, materials, and services	33.33	16.67	66.67
Standard 3: Enable and train staff for personal and organizational health literacy	28.57	33.33	73.81
Sub-standard 3.1 (14 items): Personal and organizational health literacy is understood as an essential professional competence for all staff working in the organization	28.57	33.33	73.81
Standard 4: Provide and support easy navigation and access to documents, materials, and services	48.21	52.35	67.67
Sub-standard 4.1 (9 items): The organization enables first contact via an user-friendly website and phone	44.44	48.15	59.26
Sub-standard 4.2 (5 items): The organization provides information necessary for patients and visitors for getting to the organization	100.00	86.67	73.33
Sub-standard 4.3 (14 items): Support is available to help patients and visitors to navigate the hospital	42.86	35.71	71.43
Sub-standard 4.4 (6 items): Health information for patients and visitors is available for free	5.56	38.89	66.67
Standard 5: Apply health literacy best practices in all forms of communication with patients	61.97	45.96	73.35
Sub-standard 5.1 (14 items): Verbal communication with patients is of high quality and easy to understand	38.10	35.71	57.14
Sub-standard 5.2 (5 items): Written materials are of high quality, easily accessible, and easy to understand	66.67	53.33	80.00
Sub-standard 5.3 (4 items): Digital services and new media are of high quality, easily accessible, and easy to use	41.67	0.00	58.33
Sub-standard 5.4 (9 items): Information and communication is offered in the languages of relevant patient groups by specific, trained personnel and for all provided materials	92.59	74.07	96.30
Sub-standard 5.5 (8 items): Communication that is easy to understand and to act on, especially in high-risk situations, is accepted as a necessary safety measure	70.83	66.67	75.00
Standard 6: Promote personal health literacy of patients and relatives after discharge	49.83	66.84	63.80
Sub-standard 6.1 (6 items): The organization supports patients in improving health literacy with regard to the self-management of specific health conditions	66.67	61.11	72.22
Sub-standard 6.2 (3 items): The organization supports patients in improving health literacy with regard to the development of more healthy lifestyles	22.22	66.67	55.56
Sub-standard 6.3 (11 items): Upon discharge, patients are well informed about their future treatment and recuperation process	60.61	72.73	63.64
Standard 7: Promote personal health literacy of staff with regard to occupational risks and personal lifestyles	80.00	73.33	73.33
Sub-standard 7.1 (10 items): The organization supports staff in improving their knowledge and skills for the self-management of occupational health, safety risks, and healthy lifestyles	80.00	73.33	73.33
Standard 8: Contribute to promoting personal and organizational health literacy in the region	10.00	0.00	55.56
Sub-standard 8.1 (3 items): The organization contributes to the improvement of personal health literacy of the local population	0.00	0.00	77.78
Sub-standard 8.2 (5 items): The organization supports the dissemination and further development of organizational health literacy in the geographic region and beyond	20.00	0.00	33.33
Total	41.62	39.15	63.22

The two local hospitals presented similar results, with a degree of fulfillment generally lower than the academic hospital: the latter achieved the highest overall level of OHL responsiveness, with a score of 63.22%, followed by Local Hospital 1 (41.62%) and Local Hospital 2 (39.15%). The highest degree of fulfillment was obtained for Standard 7, which measures the promotion of personal Hl of the staff concerning occupational risks and personal lifestyles (score for Local Hospital 1: 80.00%; score for Local Hospital 2: 73.33%; score for academic hospital: 73.33%).

Differences between the hospitals are particularly evident for Standards 1, 2, 3, and 8. Standard 1 is the foundational precondition for the implementation of HL practices across all organizational structures and processes. In this standard, the academic hospital scored 57.94%, while the local hospitals scored 24.44% and 18.57%, respectively. Notably, scores were lower for Substandard 1.1, which focuses on the commitment of hospital management to implementing, monitoring, and improving OHL. Here, the academic hospital achieved 40.00%, while both local hospitals scored 6.67%. On the other hand, scores were higher for the remaining two substandards, although the largest discrepancy between local and academic hospitals was observed in Substandard 1.3, which measures the quality management of OHL interventions. For Standard 2, which assesses the codevelopment of documents, materials, and services with stakeholders, the academic hospital again outperformed the local hospitals (66.67% vs. 26.67% and 18.33%, respectively). While the involvement of patients in codevelopment (Substandard 2.1) was consistent across the local hospitals, there was more variation in staff involvement (Substandard 2.2), with Local Hospital 1 scoring 33.33% and Local Hospital 2 scoring 16.67%. For Standard 3, which evaluates staff training in HL, the academic hospital presented the highest score as well, with high differences compared with the local hospitals (73.81%, 28.57%, and 33.33%, respectively). Moreover, the academic hospital was the only one to fulfill Standard 8 (score for Local Hospital 1: 10.00%; score for local hospital: 0.00%; score for academic hospital: 55.56%), which investigates the promotion of personal HL within the local population (Substandard 8.1) and the dissemination of OHL in the served (Substandard 8.2). An important gap was also noted in Substandard 8.1 (score for Local Hospital 1: 0.00%; score for Local Hospital 2: 0.00%; score for academic hospital: 77.78%), while scores were more consistent for Substandard 8.2.

For Standards 4 (Provide and support easy navigation and access to documents, materials, and services), 5 (Apply HL best practices in all forms of communication with patients), 6 (Promote personal HL of patients and relatives after discharge), and 7 (Promote personal HL of staff with regard to occupational risks and personal lifestyles), consistent results were observed across the three hospitals, although relevant differences were raised while considering some substandards. For example, Substandard 5.3 (Digital services and new media are of high quality, easily accessible, and easy-to-use) ranged from 0% to 58.3%; Substandard 6.2 (The organization supports patients in improving HL regarding the development of more healthy lifestyles) ranged from 22.22% to 55.56%.

## DISCUSSION

### General discussion of the results and comparison with previous empirical findings

In 2023, the Steering Committee for Human Rights in the fields of Biomedicine and Health of the Council of Europe published the “Guide to health literacy—Contributing to trust building and equitable access to healthcare.” The guide is intended for “decision-makers, health professionals and health providers to help them identify the needs of individuals in accessing healthcare, and to undertake corresponding responsibilities in healthcare, disease prevention and health promotion” (COE 2024). In that guide, it is stressed that to ensure equitable access to healthcare, there is the need for improving the HL of both people and systems, to strengthen the equity of opportunities in accessing healthcare. In fact, the adoption of a systematic OHL approach can help improve healthcare delivery by making information and services easier to navigate, reducing HL challenges for patients, staff, and the broader community. From this perspective, the OHL project—implemented within the WHO M-POHL action network—can play a pivotal role in initiating and facilitating the self-assessment of OHL within healthcare organizations, using a validated, recognized, and widely shared tool. In line with Ottawa Charter principles, the OHL project uses a settings approach with a triple focus—healthcare, disease prevention, and health promotion (Levin-Zamir and Straßmayr 2024)—which enlarges the perspective of hospital operability and functions to health aspects different from “traditional” diagnosis and care.

As part of the Italian participation in the WHO M-POHL action network, we evaluated the extent to which OHL principles are implemented in two local hospitals and one academic hospital, using the OHL-Hos tool, previously translated and culturally adapted for our country. To the best of our knowledge, no other studies have been published that report hospital evaluation results using the same tool, limiting the possibility of direct comparison.

The results show that the OHL principles explored by the tool are only partially implemented, with differences between the local (generally less health literate) and the academic hospitals. It is evident that a specific policy guided by the chief management is lacking in local hospitals. This kind of policy aims to implement OHL best practices across all structures and processes of the organization. Furthermore, it would involve all stakeholders in the development of patient- and staff-oriented documents and materials. The results are in line with what emerged from a previous study conducted in the hospitals located in the same geographical area using a different evaluation tool (Bonaccorsi *et al*. 2020). Specifically, in that study, it emerges that: some differences between types of hospitals are significant (accredited private hospitals were be more health literate, followed by the teaching and then the local public hospitals), and the issues with worse situations seem to be those related to the explicit integration of HL into management practices and those related to the training of the employees in HL.

Nevertheless, in the three hospitals some areas are well implemented, presumably because they are linked to other conceptual frameworks or regulatory principles with a more established “tradition” in Italy—such as quality and safety, prevention of occupational risks, and health promotion of the staff—or are more broadly embedded into practices. The latter aligns with the findings of a previous study conducted in Italy using a different assessment tool, in which the lower degree of fulfillment of OHL principles stemmed from the incorporation of HL into management practices, while the enhanced compliance was attributed to the exchange of information and communication between professionals and patients (Bonaccorsi *et al*. 2020). A paucity of involvement of different stakeholders—patients *in primis*—in the development of materials and pathways was also observed in Germany (Häberle *et al*. 2024).

Stakeholders’ involvement in developing and sharing documents, materials, and services in a participatory manner is challenging. A recent systematic review showed that patient involvement at the system level remains slow worldwide (Miller and Reihlen 2023). Case studies from Norway, Taiwan, and Denmark highlight that regulatory authorities can accelerate progress through patient rights legislation, financial incentives, and national information frameworks (e.g. decision aids, clinical guidelines, and staff training) (Miller and Reihlen 2023). National coordination of resources and guidelines is recommended to enhance patient involvement, with benefits such as cost savings, improved healthcare quality, HL, and patient autonomy. Implementation of stakeholders’ involvement at the provider level is also slow due to economic pressures, staff attitudes, and inadequate training (Miller and Reihlen 2023). Co-design documents, materials, and services with patients and other staff members (including workforce) could help in understanding local HL strengths, needs, and preferences and build on community assets by strengthening the competencies of trusted community influencers, leaders, and connectors. From this perspective, co-design allows targeting communication strategies and pathways to address the HL needs of groups of people who are encountering barriers to accessing health information and healthcare due to their limited skills (Osborne *et al*. 2022). Moreover, many studies highlighted that using participatory methods to engage health service users in the development of documents and resources is possible and effective, and that enhances trust in the institution, although it could be time-consuming (Haldane *et al*. 2019, Moore *et al*. 2019, Bird *et al*. 2021, Wiles *et al*. 2022).

The lowest degree of fulfillment of OHL principles is shown for the Standard 8 “Contribute to promoting personal and OHL in the region,” especially for local hospitals. In fact, in Italy, local hospitals are part of local health units, in which other services have the responsibility to improve the HL of the local population and of the healthcare workforces, as well as to develop OHL in the geographic region; for this reason, hospitals have a more patient-centered approach rather than a population-centered one. In contrast, the academic hospital is a healthcare organization that integrates clinical care, medical education, and research, with a national-level perspective. It is affiliated with a university, providing advanced medical services, training for healthcare professionals, and conducting medical research, while serving as part of the national healthcare system. In this light, the higher degree of fulfillment of OHL principles compared with local hospitals was expected, in line with what has been already described in other studies conducted in Italy that used different assessment tools (Bonaccorsi *et al*. 2020), but contrasting from studies performed elsewhere (Häberle *et al*. 2024, Qin *et al*. 2024).

Future assessments of OHL levels in other healthcare services other than the hospital—such as primary care and the prevention departments —could help determine whether these aspects are adequately fulfilled within the Italian healthcare system. In fact, increased efforts should be made to assess the extent to which OHL can contribute to health promotion and disease prevention services (Palumbo 2021). With regard to primary care services, a self-assessment tool called “Organizational Health Literacy in Primary Health Care Services” (OHL-PH) has been developed and is currently being tested. It was developed within the OHL project of the WHO M-POHL network and is based on the OHL-Hos.

### Implications for strengthening organizational health literacy

Our results indicate the need for targeted interventions to ensure that hospital management and leadership—particularly in local hospitals—recognize the importance of implementing the principles of OHL, and to support them with national and regional policies.

Implementing effective OHL interventions in real-world contexts requires a systematic approach. In a recent review, Meggetto *et al*. (2020) synthesized the factors influencing the operationalization of OHL principles. According to the authors, accreditation, policy drivers, executive leadership, and a culture of quality improvement provided the appropriate context for effective interventions; moreover, they found that the dominant mechanism influencing the implementation of OHL interventions were staff knowledge of OHL, internal HL expertise, and shared responsibility. From this perspective, healthcare managers and leadership play a pivotal role in building health literate organizations. In fact, HL leadership and health literate workforce are fundamental parts of health system’s capacity, ensuring that the conditions are in place to achieve health improvement, and that systemic effort can be multiplied and sustained over time, independent of external events (Sørensen *et al*. 2021).

In his review, Pelizzari *et al*. (2025) identified several key facilitators for the implementation of OHL, with team participation and engagement emerging as the most commonly cited. This underscores the value of collaborative approaches that involve a range of stakeholders. The study also emphasizes the importance of HL training and the integration of HL practices into everyday routines, highlighting the need for ongoing professional development. Clear and accessible communication—both written and verbal—is essential in supporting HL, while leadership support plays a critical role in advancing HL initiatives. Additional facilitators include strategic planning, the incorporation of OHL, and patient-centered principles into institutional policies, and the provision of easy access and navigation within services. Overall, the findings affirm that successful OHL implementation requires a comprehensive strategy that combines strong leadership, clear communication, structured planning, and continuous training.

### Strengths and limitations

This study has strengths and limitations.

Among the strengths, it represents a comprehensive assessment of hospital OHL with a tool that investigates different areas and dimensions of OHL. Moreover, we have explored the OHL of both general local hospitals and a national academic hospital, whose patients are more complex, the number of hospital beds is higher, and the management of the whole structure requires specific training and skills.

Among the limitations, we are aware that three facilities are not representative of the OHL of a region or a nation. Moreover, the study follows a cross-sectional design, which precludes the verification of future expectations, as the culture of HL and OHL gradually became established in various healthcare settings. Moreover, interrater reliability has not been measured.

Despite these limitations, measuring OHL with a tool used internationally should allow for comparisons and help identify critical areas where interventions to improve OHL are needed.

## CONCLUSION

OHL principles were only partly addressed within the hospitals included in this investigation. What seems to emerge is a need for improvement in many areas of OHL, which could produce higher-quality services for the population, considering that a better organization underpins better processes of diagnosis, care, prevention, and health promotion. The final aim of OHL is, in fact, to produce more valuable outcomes in terms of people’s health, which represents the first and foremost objective of any healthcare system. The OHL-Hos tool proved to be useful in indicating the most critical areas needing *ad hoc* interventions. The participation of the hospital working groups in the OHL-Hos assessment raised interest toward this matter and testifies that the way to a mature dimension of public HL can—and hopefully must—involve hospitals. In fact, the self-assessment process fostered a critical analysis and an in-depth review of specific organizational aspects of the hospital, in light of HL principles. From this perspective, we believe that the evaluation itself can be considered an initial intervention aimed at promoting the adoption of HL-oriented practices within hospital settings.

## Data Availability

The data underlying this article will be shared on reasonable request to the corresponding author.
