# Correction to: Insights into organizational health literacy in Italian hospitals: findings from the M-POHL network project

**DOI:** 10.1093/heapro/daag108

**Published:** 2026-08-04

This is a correction to: Chiara Lorini, Luigi Palmieri, Brigid Unim, Salvatore Zimmitti, Carla Lunetta, Claudia Biagi, Francesco Toccafondi, Patrizio Zanobini, Simone Iadevaia, Maria Gabriella Cacciuttolo, Camilla Lombardo, Benedetta Marcozzi, Angela Ancona, Andrea Paladini, Daniela Galeone, Maria Lucia Specchia, Guglielmo Bonaccorsi, Insights into organizational health literacy in Italian hospitals: findings from the M-POHL network project, *Health Promotion International*, Volume 40, Issue 4, August 2025, daaf137, https://doi.org/10.1093/heapro/daaf137

In the originally published version of this manuscript, the introduction erroneously stated that the International Self-assessment Tool for Organizational Health Literacy (of Hospitals (OHL-hos) was developed by M-POHL.

The relevant sentence has been corrected to read:

“As a goal of the Project 2, and based on the V-HLO framework, M-POHL piloted the International Self-assessment Tool for Organizational Health Literacy (responsiveness) of Hospitals (OHL-Hos), through a participatory process (International Working Group Health Promoting Hospitals and Health Literate Healthcare Organizations 2019, Finbraten et al. 2024)”.

Instead of:

“As a goal of the Project 2, and based on the V-HLO framework, M-POHL developed the International Self-assessment Tool for Organizational Health Literacy (responsiveness) of Hospitals (OHL-Hos), through a participatory process (International Working Group Health Promoting Hospitals and Health Literate Healthcare Organizations 2019, Finbraten et al. 2024)”.

